# YciR, a Specific 3′-Phosphodiesterase, Plays a Role in the Pathogenesis of Uropathogenic *Escherichia coli* CFT073

**DOI:** 10.3389/fmicb.2022.910906

**Published:** 2022-07-18

**Authors:** Si Zhang, Jingting Wang, Yu Fan, Wang Meng, Chengqian Qian, Peng Liu, Yi Wei, Chao Yuan, Yuhui Du, Zhiqiu Yin

**Affiliations:** ^1^Ministry of Education (MOE) International Joint Research Laboratory on Synthetic Biology and Medicines, School of Biology and Biological Engineering, South China University of Technology, Guangzhou, China; ^2^College of Life Science, Nankai University, Tianjin, China; ^3^Tianjin First Central Hospital, Tianjin, China; ^4^Department of Sanitary Toxicology and Chemistry, School of Public Health, Tianjin Medical University, Tianjin, China; ^5^National Engineering Research Center for Efficient Utilization of Soil and Fertilizer Resources, College of Resources and Environment, Shandong Agricultural University, Tai’an, China

**Keywords:** uropathogenic *Escherichia coli*, *yciR*, phosphodiesterase, cyclic dimeric guanosine monophosphate, urinary tract infections

## Abstract

Urinary tract infections (UTIs), with the characteristics of recurrence and resistance to antibiotics due to misuse, remain a common health and economic issue for patients. Uropathogenic *Escherichia coli* (UPEC), which is capable of evading the immune response by forming intracellular bacterial communities (IBCs) in the cytoplasm of bladder epithelial cells (BECs) after invasion, has been shown to be the prevailing cause of UTIs. Cyclic dimeric guanosine monophosphate (c-di-GMP) is a small molecule responsible for eliciting the innate immune response of the host only if it has not been degraded by some phosphodiesterases (PDEs), such as YciR. The relationship between YciR and c-di-GMP levels in UPEC is inconclusive. In this study, we investigated the gene expression profile of UPEC in BECs and identified *yciR* as an upregulated gene. Western blot revealed that YciR enhanced the virulence of UPEC by inhibiting the phosphorylation of NF-κB. The expression of *yciR* could be repressed by HupB in a directly binding manner. We identified YciR, a novel PDE, and defined its possible function in innate immune evasion. We also demonstrated that YciR is an HupB-dependent PDE that degrades c-di-GMP and that a low concentration of c-di-GMP might make NF-κB less phosphorylated, thereby reducing the host’s pro-inflammatory response. This is the first time that YciR has been identified as a virulence factor in the pathogenesis of UPEC. These findings further increase our understanding of the pathogenesis of UPEC and provide a theoretical basis for further studies.

## Introduction

Urinary tract infections (UTIs) are one of the most frequently diagnosed illnesses in hospitals and communities. Over 85% of the reported UTI cases, especially complicated ones, are associated with uropathogenic *Escherichia coli* (UPEC) (Ulett et al., 2013). Women seem to be more susceptible to UTIs than men due to their anatomy; however, age, genetics, sexual intercourse, and urological factors also influence susceptibility to UTIs (Sivick et al., 2010). It is estimated that 40% of women experience at least one UTI in their lifetime (Terlizzi et al., 2017). Infection by UPEC is mainly initiated in a manner of colonization in the bladder epithelium or urinary tracts (Ragnarsdóttir et al., 2011). Upon entering the epithelium, UPEC breaks the fusiform vesicle and begins to replicate into biofilm-like intracellular bacterial communities (IBCs) in the cytoplasm, which are essential for the establishment of acute and chronic infections (Justice et al., 2004; Engel et al., 2006). The expression of type I fimbriae enables UPEC to attach to bladder epithelial that express mannosylated uroplakins (Behzadi, 2020; Sarshar et al., 2020; Behzadi et al., 2021b). The formation of IBCs can also help UPEC evade host immune defenses, such as cytokines secreted by the host or the migration of immune cells, including neutrophils (Haraoka et al., 1999). Key transcription factors, such as NF-κB, are activated during UPEC infection, leading to innate and acquired immune-inflammatory responses (Behzadi et al., 2022).

With a variety of mediators, complexes, and signaling pathways, Toll-like receptors (TLRs), which are transmembrane proteins, can protect the host’s urinary tract from harmful microbes, such as UPEC, which can cause UTIs. The most competent molecules in the urinary tract defense mechanism are TLR2, TLR4, and TLR5 (Behzadi and Behzadi, 2016; Behzadi et al., 2021a). In addition, innate immunity is a primary defense mechanism to effectively clear extracellular and intracellular UPEC with the help of TLR4 molecules of the bladder (Abraham and Miao, 2015). Cyclic dimeric guanosine monophosphate (c-di-GMP) is a small molecule found in bacteria, not in human beings or other mammals (Cui et al., 2019), initially demonstrated to regulate cellulose synthesis in *Acetobacter xylinum* (Ross et al., 1987). Accumulating evidence indicates that c-di-GMP can be produced by pathogens and can trigger the immune response of the host (Ding et al., 2020). The following two direct cytosolic receptors for c-di-GMP have been characterized: STING and DDX4 (Bowie, 2012). Furthermore, emerging reports have revealed that this second messenger also regulates the host’s response. McWhirter et al. (2009) found that c-di-GMP is capable of activating NF-κB, and Kobayashi et al. (2015) demonstrated that the effects of c-di-GMP were fully restored when NF-κB was inhibited in hematopoietic stem and progenitor cells (HSPCs). In addition, c-di-GMP has been shown to be an immunomodulatory molecule that is highly immunogenic and produces a higher antibody response in vaccinated mice than in those administered the antigen only (Karaolis et al., 2007). C-di-GMP is produced by diguanylate cyclases and can be degraded by specific phosphodiesterases (PDEs) such as YciR. YciR has been reported as a specific 3′-PDE with an EAL domain, and it has a GGDEF domain of unknown function (Weber et al., 2006). C-di-GMP is a ubiquitous second messenger that impacts bacteria motility and biofilm formation (Lindenberg et al., 2013). During infection, bacteria can release c-di-GMP or c-di-AMP to the host, acting as pathogen-associated molecular patterns (PAMPs) to trigger the host’s innate immune response (Hengge, 2009; Jiang et al., 2017). Furthermore, c-di-GMP has been demonstrated to be involved in many functions of bacteria, such as bacterial adhesion, colonization, and biofilm formation (Rashid et al., 2003; Karaolis et al., 2005). YciR has also been demonstrated to upregulate *csgD* expression, which leads to rdar biofilm morphotype development as a virulence gene in UPEC (Behzadi and Behzadi, 2017; Cimdins et al., 2017). However, there is seldom a discussion on the relationship between the virulence of YciR and the pathogenesis of UTIs.

In this study, we combined the high-throughput transcriptome similar to that which has been carried out to identify and determine the regulation of host responses to bacterial infection (Ranjbar et al., 2017), molecular biology methods, cell line culture methods, and a mouse model of UPEC bladder cystitis to further investigate the exact role of YciR in the innate response of bladder epithelial cells (BECs) facing the invasion and infection of UPEC. CFT073 is one of the well-studied uropathogenic *E. coli* strains, according to a previous study related to the UPEC transposon library; thus, we chose this prototype strain as the representative strain in our investigation (Shea et al., 2020). Our results suggest that c-di-GMP is a stimulator of the host’s antibacterial response. *yciR*, which is upregulated by HupB, degrades the level of c-di-GMP to inhibit the phosphorylation of NF-κB to enable the colonization of UPEC.

## Results

### Overview of Gene Expression in the Transcriptome

**FIGURE 1:**
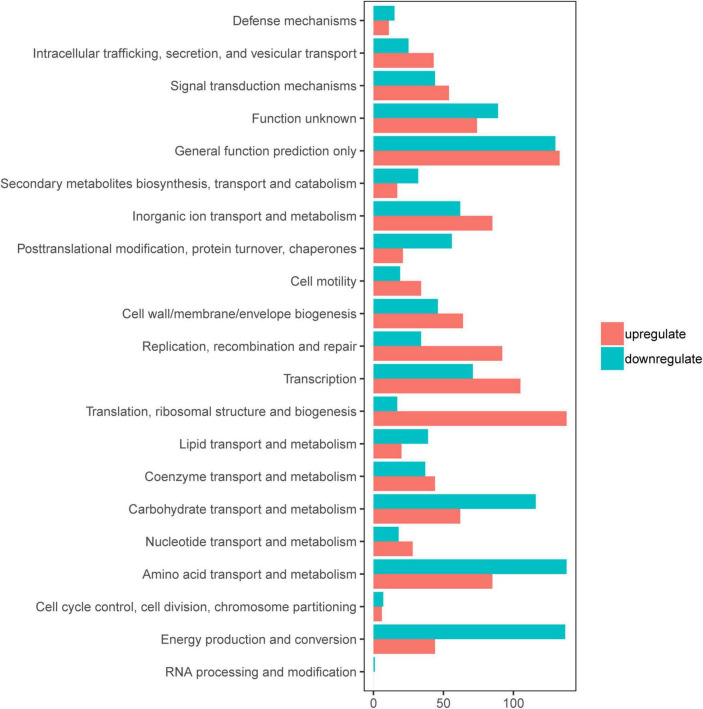
Clusters of orthologous groups (COGs) analysis of differentially expressed genes (DEGs) in *E. coli* CFT073 after 8 h of invasion of the 5637 bladder cell line. Red bars indicate upregulated genes, while blue bars indicate downregulated genes.

To gain an understanding of *E. coli* CFT073′s fitness for the pathogenesis of UTIs, the transcriptome of *E. coli* CFT073 cultured intracellularly in 5637 bladder cell line compared with those cultured in Luria-Bertani (LB) broth was analyzed. Differentially expressed genes (DEGs) were further investigated. Genes responsible for the virulence of UPEC, such as adhesion, iron acquisition system, and other reported virulence factors (Subashchandrabose and Mobley, 2015; Behzadi and Behzadi, 2017), were found to have great fold changes in the progress of *E. coli* CFT073 infection (Table 1). The transcriptomic results were validated by selecting a subset of genes for RT-PCR analysis. For RT-PCR analysis, we randomly chose five upregulated and five downregulated genes that showed statistically significant fold changes in the mRNA expression profile. The trend was consistent with the transcriptome, although the fold-change varied from the mRNA expression profiling, presumably because qPCR is generally more sensitive to expression differences than the transcriptome (Supplementary Figure 1). According to the screening criteria, 2,401 genes were differentially expressed during *E. coli* CFT073 survival in BECs (Supplementary Figure 2). In addition, the DEGs were clustered within 20 Clusters of Orthologous Groups (COGs) function categories based on the classification of NCBI (Figure 1). The upregulated genes were significantly enriched in the “translation ribosomal structure” and “biogenesis” categories, indicating that UPEC needs to synthesize proteins similar to some virulence factors for infection. Remarkably, UPEC had downregulated genes enriched in the transport and metabolism of carbohydrates and amino acids, and energy production categories, suggesting that UPEC might downregulate its metabolism in the niche of BECs. This indicates that *E. coli* CFT073 may alter specific gene expressions to adapt to the environment in BECs.

**TABLE 1:** Genes positively regulated 8 h postinfection.

Gene name	Fold change	Function
*yadC*	21.2	Fimbrial protein
*fimD*	4.83	Outer membrane usher protein fimD precursor
*fimB*	5.37	Type I fimbriae Regulatory protein fimB
*fimC*	3.13	Chaperone protein fimC precursor
*fimH*	3.4	FimH protein precursor
*chuA*	37.48	Outer membrane heme/hemoglobin receptor
*chuS*	10.43	Putative heme/hemoglobin transport protein
*chuT*	15	Putative periplasmic binding protein
*sat*	16.95	secreted auto transporter toxin
*sitA*	53.96	SitA protein
*sitB*	11.48	SitB protein
*sitC*	4.58	SitC protein
*sitD*	3.64	SitD protein
c2931	4.51	Manganese transport protein mntH
*tar*	5.26	Methyl-accepting chemotaxis protein II
*tsr*	9.14	Methyl-accepting chemotaxis protein I
*rpoE*	2.42	RNA polymerase sigma-E factor
*glnA*	3.43	Glutamine synthetase
*iroB*	9.29	Putative glucosyltransferase
*yhjX*	3.5	Protein yhjX

### yciR Is Upregulated in the Transcriptome and Contributes to *Escherichia coli* CFT073, the Colonization of Bladder Epithelial Cells

**FIGURE 2:**
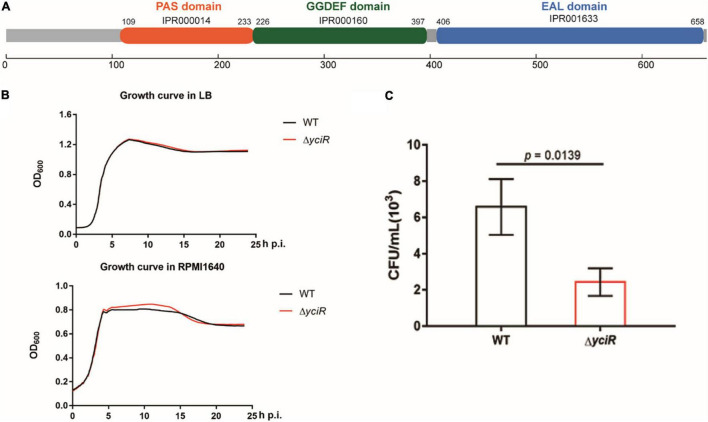
Features of YciR. **(A)** Domain identification of YciR. **(B)** Growth curves of WT and Δ*yciR*. Growth curves of WT and Δ*yciR* strains cultured in LB broth. Growth curves of the WT and Δ*yciR* strains cultured in RPMI 1640. The overnight cultured strains were diluted at a ratio of 1:1,000 and then cultured under different conditions in LB broth or RPMI 1640. The spectrophotometric value was measured at OD_600_ every 10 min. **(C)** *yciR* impacted the invasion of BEC infection. The 5637 bladder cell line was seeded in 24-well plates and then infected with WT and *yciR* mutant strains for 8 h. CFU counts of triplicate wells were representative of three independent experiments (two-tailed *t*-test).

*yciR* encodes a putative 3′-PDE with a 4.35-fold upregulation in BECs (Weber et al., 2006). YciR in *E. coli* CFT073 comprised 661 amino acids. According to InterPro annotation, this sequence contained a PAS domain (IPR000014), a GGDEF domain (IPR000160), and an EAL domain (IPR001633) (Figure 2A). However, the biological function of YciR in UPEC has not yet been studied. To determine whether *yciR* influences the virulence of UPEC to survive in BECs, we first compared the growth curves of the *E. coli* CFT073 and *yciR* mutant strain in LB broth and RPMI 1640 medium. These two strains exhibited similar growth curves under *in vitro* conditions (Figure 2B), demonstrating that YciR was not essential for the growth of *E. coli* CFT073. Wild type (WT) and Δ*yciR* were then used to infect the 5637 bladder cell line for 2 h, followed by a gentamicin protection assay for another 6 h. Δ*yciR* exhibited a decreasing ability to invade the 5637 bladder cell line compared with the WT (Figure 2C, *p* = 0.0139). These results indicate that YciR plays an important role in the colonization of the BECs.

### *yciR* Is Involved in Acute Bladder Infections

**FIGURE 3:**
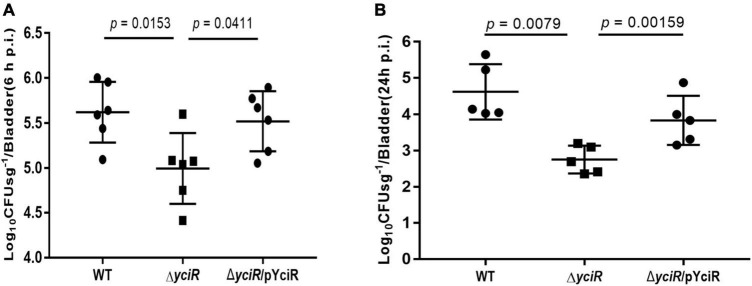
*yciR* promotes *E. coli* CFT073 colonization in the bladders of Balb/c mice in acute infections. **(A)** Determination of the transurethral colonization ability of WT, Δ*yciR*, and Δ*yciR*pYciR strains in the bladders of Balb/c mice at 6 hpi. **(B)** Determination of transurethral colonization ability of WT, Δ*yciR*, and Δ*yciR*pYciR strains in the bladders of Balb/c mice at 24 hpi. The homogenates from the infected bladders of Balb/c mice with WT and *yciR* mutant strains were plated on the agar for CFU counts. The bars in the graph represent the mean ± *SD*. Each dot represents the bladder of one mouse. Three independent experiments were performed, and *p*-values were calculated using a two-tailed Mann–Whitney non-parametric comparison.

**FIGURE 4:**
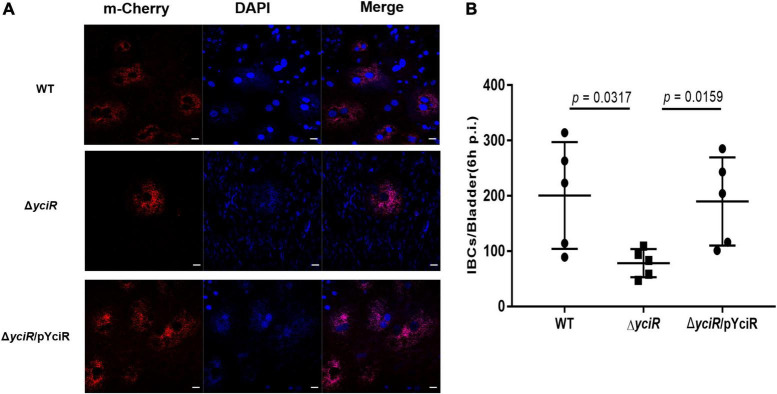
YciR promoted the number of IBC formations in Balb/c mouse bladders. **(A)** Microscopic examination of IBC formation in mice bladders infected with WT, Δ*yciR*, and Δ*yciR*pYciR strains in Balb/c mice killed at 6 hpi after transurethral catheterization. The harvested bladders were prepared as described in the Materials and Methods section for visualization by confocal laser scanning microscopy. Observation of intracellular WT, Δ*yciR*, and Δ*yciR*pYciR (red) associated with the bi-nucleus of superficial umbrella cells of the bladder (blue). Bar = 20 μm. **(B)** IBC enumeration in Balb/c mice bladders infected by the WT, Δ*yciR*, and Δ*yciR*pYciR strains. The number of IBCs was counted *via* confocal laser microscopy for the whole bladder. A single Z-section of a superficial umbrella BEC of Balb/c revealed an IBC using confocal laser microscopy. Each dot represents the IBC number in one mouse bladder. Three independent experiments were performed, and *p*-values were calculated using a two-tailed Mann–Whitney non-parametric comparison.

To explore the role of YciR in acute UTIs, female Balb/c mice were infected transurethrally with 10^7^ CFU of WT or Δ*yciR* strains. Bacterial titers in bladders infected by Δ*yciR* at 6 h and 24 h pi were significantly lower than those in bladders infected by the WT (Figure 3A, *p* = 0.0153, and Figure 3B, *p* = 0.0079) and were restored in Δ*yciR*pYciR-infected mice (Figure 3A, *p* = 0.0411, and Figure 3B, *p* = 0.00159). This suggests that YciR affects the colonization of UPEC in the bladders of mice. Moreover, UPEC can invade urinary superficial epithelial cells and form IBCs clonally to evade host defenses. We then investigated the number of IBCs in mice to evaluate acute bladder infections. The mCherry-expressing bacteria were counted by confocal microscopy (Figure 4A). The number of IBCs was significantly lower in the *yciR* mutant strain compared to the WT (Figure 4B, *p* = 0.0317), and the number of IBCs was restored in Δ*yciR*pYciR-infected mice (Figure 4B, *p* = 0.0159). These results show that YciR deficiency impairs UPEC colonization in the bladder and IBC formation.

### *yciR* Enhances the Colonization of the Bladder Epithelial Cells by Suppressing NF-κB

**FIGURE 5:**
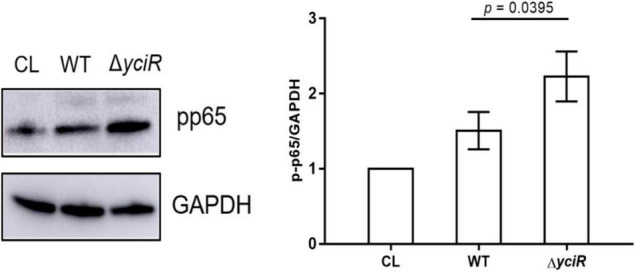
Invasion with the WT and *yciR* mutant strains induced a difference in the 5637 bladder cell line to increase the phosphorylation of NF-κB. The 5637 bladder cell line was infected with WT and *yciR* mutants for 8 h, and p-p65 was used to measure NF-κB phosphorylation. GAPDH was used as the loading control. CL refers to cell lysis for the blank control. The error bars represent the mean ± *SD*, and three independent experiments were performed (two-tailed *t*-test).

The c-di-GMP has been implicated in the activation of NF-κB (McWhirter et al., 2009). Meanwhile, YciR has been proven to contain an EAL domain that decreases the c-di-GMP produced by *E. coli* (Lindenberg et al., 2013). Phosphorylation of NF-κB initiated a series of downstream signal events for a proinflammatory response (Zhang et al., 2006). To explicate the mechanism for decreased virulence in the *yciR* mutant, we analyzed phosphor-p65 of NF-κB after an invasion of WT and Δ*yciR* strains by Western blot. Increased NF-κB phosphorylation was observed in Δ*yciR* compared to the WT strain (Figure 5, *p* = 0.0395), suggesting that the reduction level of phosphorylated NF-κB is important for the colonization of *E. coli* CFT073.

### Expression of *yciR* Is Repressed by the Regulator HupB in the Pathogenic Processes of Uropathogenic *Escherichia coli*

**FIGURE 6:**
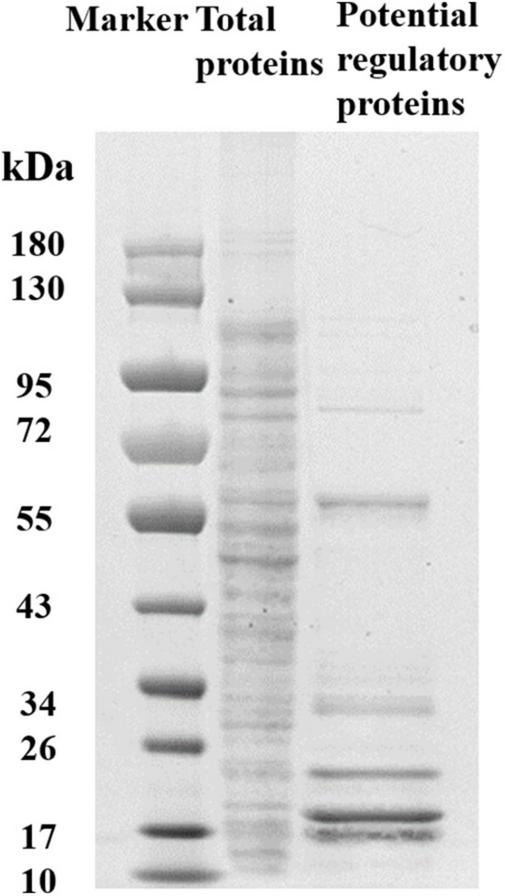
SDS-PAGE identification of DNA pulldown using the promotor region of *yciR*. M, Marker; lane 1, totally bacterial lysis; lane 2, potential proteins enriched by the *yciR* promotor. Using the abovementioned DNA pulldown technique, we used the assumed promotor of *yciR* as the bait to identify potential regulatory proteins.

**FIGURE 7:**
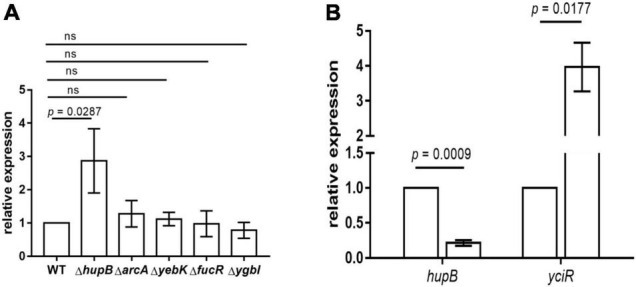
*yciR* expression in different mutant strains of *E. coli* CFT073 and the intracellular regulation of HupB. **(A)** *yciR* expression influenced by *hupB*, *arcA*, *yebK*, *fucR*, and *ygbI* mutant strains. **(B)** Intracellular expression of *hupB* and *yciR* when the 5637 bladder cell line was infected with *E. coli* CFT073. Expression was normalized with the internal control gene *gyrA.* The error bars represent the mean ± *SD*, and three independent experiments were performed (two-tailed *t*-test).

**FIGURE 8:**
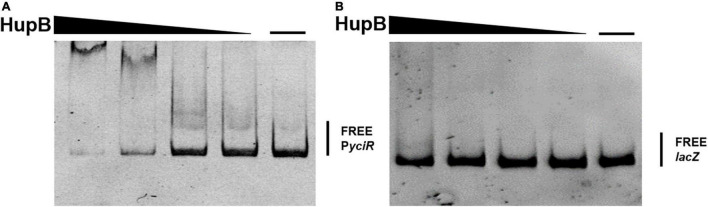
HupB directly binds to the *yciR* promotor. **(A)** EMSAs of the *yciR* promotor with the purified HupB protein. **(B)** *lacZ* fragment was used as the negative control.

To determine the regulators of *yciR* expression in UPEC, we performed a DNA-pulldown assay with the presumed promotor of *yciR* as bait to identify potential regulatory proteins (Figure 6). We identified 10 potential proteins and analyzed the possibility of regulating *yciR* expression (Table 2). Mutations in 5 of 10 candidates (*hupB*, *arcA*, *yebK*, *fucR*, and *ygbI*) showed no significant alteration in the expression of *yciR*; however, a mutant of *hupB* showed a significantly increased expression of *yciR* intracellularly (Figure 7A, *p* = 0.0287). Therefore, HupB is speculated to be a candidate for regulating *yciR* expression. We then investigated the intracellular expression of *hupB* and *yciR* and found that *hupB* was downregulated, while *yciR* was upregulated after the invasion of UPEC (Figure 7B, *p* = 0.0009 and *p* = 0.0177, respectively). We then explored the interaction between the purified HupB-His_6_ protein and the presumed promotor of *yciR* using EMSA. With increasing concentrations of HupB, slow migration bands were observed for the *yciR* promotor, while no obvious movement retardation was found for the *lacZ* fragment, which served as the negative control, indicating that HupB can directly bind to the *yciR* promotor *in vitro* (Figure 8). These results imply that HupB represses the expression of *yciR* by directly binding to its promotor.

**TABLE 2:** Potential proteins for regulating the *yciR.*

Number	Protein	Function
1	SpeG	Spermidine N (1)-acetyltransferase
2	C0307	Hypothetical protein
3	ArcA	Aerobic respiration control protein arcA
4	MinD	Septum site-determining protein minD
5	YebK	Hex regulon repressor
6	FucR	L-fucose operon activator
7	YgbI	Hypothetical transcriptional regulator ygbI
8	HupB	DNA-binding protein HU-beta
9	Dps	DNA protection during starvation protein
10	Rpsc	30S ribosomal protein S3

## Discussion

This study represents one of the signal transduction regulatory pathways in *E. coli* CFT073, which inhibits the proinflammation of the host and evades innate immunity. C-di-GMP is a ubiquitous second messenger that impacts bacterial motility and biofilm formation, and it can be degraded by specific PDEs (Lindenberg et al., 2013). C-di-GMP signaling is involved in a number of processes, including motility, cell cycle and differentiation, and pathogenicity, with biofilm formation being the most studied (Ahmad et al., 2017). Bacteria can release c-di-GMP or c-di-AMP as PAMPs to the host during infection, prompting the host’s innate immunological response (Hengge, 2009; Jiang et al., 2017). Lindenberg et al. (2013) found that YciR functions as a trigger enzyme in the c-di-GMP signaling cascade in *E. coli* biofilm control, which senses and effectively degrades c-di-GMP-produced upstream in the regulatory cascade, thereby releasing the transcriptional regulator MlrA to activate *csgD* transcription, a key biofilm regulator activating the relative genes for synthesizing amyloid curli fibers. One study investigated the contribution of c-di-GMP to adhesion to BECs using the *E. coli* CFT073 strain with deletion of individual DGC, PDE, and DGC-PDE genes. Interestingly, YciR had no effect on the adhesion to BECs (Spurbeck et al., 2012). Our study focused on the invasion of BECs. Meanwhile, our findings suggest that YciR may play an important role in the pathogenesis of UPEC within BECs. YciR promotes the upregulation of *csgD*, which leads to the development of rdar biofilms in UPEC (Cimdins et al., 2017). STM1703, the YciR homolog in *Salmonella enterica* serovar Typhimurium (*S.* Typhimurium), has apparent PDE activity and suppresses the *csgD* expression in a distinct region between −340 and −208 upstream of *csgD* (Simm et al., 2007; Ahmad et al., 2017). Furthermore, *csgD* is regulated at multiple levels by c-di-GMP signaling. STM 1703 suppressed *csgD* transcription, whereas STM 4264, an EAL domain, suppressed *csgD* by acting on post-transcriptional processes (Ahmad et al., 2017). Simm et al. (2009) also demonstrated that STM1344 acted upstream of STM1703 and repressed STM1703 expression. Furthermore, this is the first time that YciR has been identified as a virulence factor involved in the pathogenesis of *E. coli* CFT073. The Δ*yciR* strain showed a significant deficiency in colony counting *in vitro* and *in vivo*, indicating that YciR is a new virulence factor involved in the pathogenesis of UTIs. This result is consistent with a previous study, which found that intracellular *S.* Typhimurium has three redundant PDEs that are responsible for lowering c-di-GMP to maintain intracellular survival and full virulence (Petersen et al., 2019). However, there is a contradiction in the results of the invasion of the STM2123 mutant with a dysfunctional EAL domain in *S.* Typhimurium (Ahmad et al., 2011). One possible explanation was that there were many factors that affect the invasion of *S.* Typhimurium, such as the effectors secreted from the type III secretion system, which were absent in UPEC strains (Galán, 2001). The underlying mechanisms need to be established in the future.

Intracellular UPEC is exposed to a variety of stimuli during BECs invasion and intracellular survival; however, less is known about how c-di-GMP modulates innate immunity during the critical stage of infection. In this study, we described the contribution of YciR to the reduction of c-di-GMP in promoting the survival of UPEC in BECs. YciR was able to significantly alter the phosphorylation of NF-κB after infecting BECs. It has been demonstrated that type I pilus can induce apoptosis by quantitatively suppressing NF-κB in the pathogenesis of UTIs (Klumpp et al., 2001). This indicates that there is another mechanism by which UPEC confronts the complex environment of the host to repress the NF-κB pathway, further confirming that YciR plays an important role in UTI pathogenesis, depending on the phosphorylation of NF-κB. NF-κB is one of the major transcription factors for the induction of a proinflammatory response, while BECs can activate the NF-κB-associated pathway to mediate the IL-6 response during UPEC infection (Song et al., 2007). In our study, YciR represses the NF-κB pathway by reducing the concentration of c-di-GMP to promote resistance to the host immune response. As a result, the regulation of c-di-GMP is crucial for UPEC to establish an acute infection.

**FIGURE 9:**
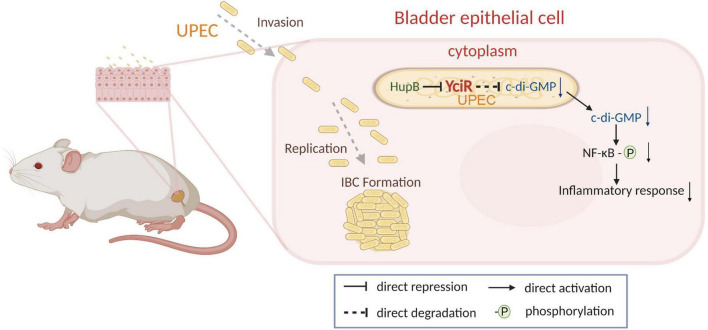
Graphical abstract of YciR-mediated virulence model of uropathogenic *Escherichia coli* CFT073.

In addition, we uncovered that YciR was directly repressed by HupB based on the DNA pulldown assay. It has been reported that the expression of *yciR* is the RopS-dependent in the *E. coil* K12 strain (Weber et al., 2006). However, *E. coli* CFT073 was deficient in the *ropS* gene, which is thought to be the result of continuing evolution and adaption to extraintestinal habitats. HupB is the β-subunit of the HU protein, which is a member of family II of the DNA binding protein (Dillon and Dorman, 2010). HupB has been reported as a critical component of the UPEC biofilm matrix (Devaraj et al., 2015). In *E. coli* CFT073, we developed a model to determine the HupB-dependent *yciR* regulation pathway and the impact of NF-κB phosphorylation (Figure 9). Careful modulation of c-di-GMP is crucial, as it can bind enzymes, riboswitches, special structure components of proteins, and transcription factors in the host (Chou and Galperin, 2016).

In this study, we discovered a specific PDE, YciR, and characterized its potential role in the evasion of innate immunity. Our study showed that YciR is an HupB-dependent PDE that degrades c-di-GMP, and a low concentration of c-di-GMP could make NF-κB less phosphorylated to reduce the proinflammatory response of the host. The role of c-di-GMP during UPEC intracellular survival has not been well understood in previous studies. To the best of our knowledge, this is the first time that a low concentration of c-di-GMP has been shown to promote the intracellular survival of UPEC. This study provides new insights into the pathogenesis of UPEC and other intracellular pathogens.

## Conclusion

The findings of this study provide critical information about the involvement of a specific PDE, YciR, which is involved in the pathogenesis of UTIs. Our *in vitro* and *in vivo* studies demonstrated that YciR is an HupB-dependent PDE that can degrade c-di-GMP and reduce the phosphorylation of NF-κB, thereby reducing the host’s proinflammatory response. A thorough understanding of these pathways is necessary to identify potential therapeutics.

## Materials and Methods

### Bacterial Strains, Plasmids, and Culture

The various strains and plasmids used in this study are listed in Supplementary Table 1. *E. coli* CFT073 (cat# 700928) was purchased from the American Type Culture Collection (ATCC) and the National Collection of Type Cultures and was used as the WT strain. The bacteria were cultured in LB broth at 37°C with shaking at 180 rpm. Appropriate antibiotics were added into the LB broth as needed, with 100 μg/ml blasticidin S (MedChemExpress, HY-K1054), 25 μg/ml chloramphenicol (Solarbio, SC9060), 50 μg/ml kanamycin (Beyotime, ST101), and 14 or 100 μg/ml gentamicin (MedChemExpress, HY-K1050) (Wright et al., 2005).

### RefSeq of CFT073 and the Relative Primers

The RefSeq assembly accession of CFT073 in this study was GCF_000007445.1. The mutant strains were constructed by disruption of the target gene *via* insertion of a chloramphenicol cassette using the previously described approach of a λ-red recombinase system (Derous et al., 2011). The complementary strain was constructed by cloning *yciR* and its own promotor to the pBluescript II-SK (+) plasmid. The recombination plasmid pET28a-*hupB* was constructed by cloning *hupB* into the pET28a plasmid. The primer sequences used for the generation and conformation of the mutant strain and other relative primers are listed in Supplementary Table 2 (Vet and Marras, 2005; Qin et al., 2009). PCR primers were designed with the Primer Premier version 5.0 (2000) to amplify the conserved regions of the target genes (Lalitha, 2000).

### Cell Culture and Invasion Assay

Human BECs were purchased from the ATCC (HTB-9). The cells were cultured in RPMI-1640 medium in addition to 10% fetal bovine serum (FBS) at 37°C in a 5% CO_2_ incubator. The BECs were seeded in 24-well plates to a confluence of almost 80%. The cells were further infected with bacteria at an multiplicity of infection (MOI) of 100, and the cell plates were centrifuged at 500 × *g* for 5 min. After 2 h of infection, the cells were washed three times with phosphate-buffered saline (PBS) to remove the unattached bacteria and then treated with 100 μg/ml gentamicin for an additional 2 h to kill the extracellular bacteria. The cells were again washed three times again with PBS, followed by another 4 h of treatment with 14 μg/ml to sustain a low concentration and kill the extracellular bacteria. The cells were again washed three times. The WT strain was lysed with 200 μl 0.1% Triton X-100 and serially diluted three times, while the *yciR* mutant strain was lysed with 100 μl of lysis buffer containing 0.1% Triton placed on LB agar (Wright et al., 2005).

### RNA Isolation Purification

Total RNA was extracted using a TRIzol Reagent (Invitrogen, #15596018) after *E. coli* CFT073 infection (Mobley, 2016). Following the extraction, eukaryotic RNA was removed using the MICROBEnrich Kit (Invitrogen, #AM1901) to enrich the remaining bacterial RNA. Then, the MICROB Express Bacterial RNA Enrichment Kit (Invitrogen, #AM1905) was used to deplete the 16S and 23S rRNA from bacterial RNA. The cDNA library was prepared using a NEBNext Ultra Directional RNA Library Prep Kit for Illumina (New England Biolabs, #E7420L).

### RNA Sequencing

RNA sequencing was performed using the Hiseq 2000 platform and analyzed by Novogene (Tianjin, China). Sequencing reads were mapped to the genome of *E. coli* CFT073 (GCF_000007445.1) (Welch et al., 2002). HTSeq version 0.6.1 was used to count the read numbers of each gene. The number of FPKM of each gene was calculated to determine the expression of each gene. The DESeq R package (1.18.0) was used to analyze the differential gene expression of *E. coli* CFT073 in BECs compared to that in LB broth, and the Benjamini and Hochberg method was used to adjust the *p*-values to control the false discovery rate (Yang et al., 2015).

### qRT-PCR Analysis

The quality of the total RNA was measured using a spectrophotometer (NanoDrop Technologies, United States). cDNA was generated using a PrimerScript RT Reagent Kit with a genome DNA eraser (TaKaRa, China) in a 1.2 μg system. qRT-PCR was performed using an ABI7500 Real-Time PCR system (Applied Biosystems, United States) to validate differential gene expressions. The reaction system was performed in a total volume of 20 μl in a 96-well optical reaction plate (Applied Biosystems, United States), which contained 10 μl SYBR Green Master Mix (Applied Biosystems, United States), 1 μl cDNA, and 1 μl of each gene-specific primer. The primers are listed in Supplementary Table 2. *gyrA*, a previously defined endogenous control gene, was used as the housekeeping gene (Takle et al., 2007), and the fold change of the target gene was determined using the 2^–ΔΔ*Ct*^ method (Santangelo et al., 2012).

### Western Blot

The bladder cell line 5637 was infected, as described in the “Cell culture and invasion assay” section. After 8 h, the cells were washed with cold PBS before lysis in RIPA buffer supplemented with 1 × complete protease inhibitor (Roche, United States). The samples were resolved by SDS-PAGE and then transferred to an immobilon PVDF membrane (Roche, United States). It was processed for Western blot analysis, as described in a previous study (Wiles et al., 2008).

### DNA-Pulldown Assay

DNA pulldown using the presumptive *yciR* promotor region was performed to identify the possible upstream regulator. Briefly, 150 bp upstream of the *yciR* coding region was amplified with biotinylated primers (Augctbio, Beijing, China). The probe was added to the cell lysates of *E. coli* CFT073 and then incubated at room temperature. The complex of promotor DNA and protein was precipitated with streptavidin-coupled DynaBeads (Thermo Fisher Scientific, China). The protein combined with DynaBeads was eluted with increasing concentrations of NaCl, ranging from 100 mM to 1 M. The protein in the elution buffer was sedimented by the trichloroacetic acid (TCA) buffer (Suzuki et al., 2010). Then, it was processed and identified using LC-MS/MS by Novogene (Tianjin, China).

### Expression and Purification of HupB

The recombinant plasmid pET28a-*hupB*, which was C-terminally tagged with 6 × His, was electronically transformed into the host strain BL21(DE3). The positive strain was cultured at 37°C in LB broth with a final concentration of 50 μg/ml kanamycin. After about 3 h of incubation, the OD_600_ reached about 0.5, and 0.1 mM Isopropyl β- d-1-thiogalactopyranoside (IPTG) was added to the culture solution to induce HupB expression at 16°C for about 12 h. After washing with PBS, the culture was resuspended with a binding buffer for ultrasound, which was composed of 50 mM Tris-HCl, 300 mM NaCl, and 10 mM imidazole. The fusion protein was then purified from the soluble extracts using a Ni-affinity chromatography column (GE Healthcare, #10280275) (Rowe and O’Gara, 2016). The protein concentration was evaluated by the Bicinchoninic Acid Assay (Sangon Biotech, #C503021) and stored at −80°C for further use.

### Electrophoretic Mobility Shift Assay

Electrophoretic mobility shift assay (EMSA) was used to detect the DNA-protein binding of HupB. The promotor of *yciR* and the negative control PCR fragments were amplified using the genomic DNA of *E. coli* CFT073 as the template. The gradient dilution of HupB from 0 to 10 μM was incubated with purified PCR fragments (20 ng) in 20 μl binding buffer (20 mM Tris-HCl pH = 7.5, 1 mM EDTA, 80 mM NaCl, 100 μg/ml BSA, and 10% glycerol) (Corcoran et al., 2010) for 30 min at room temperature. The reaction buffer was added to a 6% (w/v) native polyacrylamide gel and electrophoresed at 90 V for approximately 1.5 h at 4°C. The separated samples in the gel were then stained with 0.1% (v/v) Gel Red and visualized with a UV transilluminator (Rowe and O’Gara, 2016).

### Mouse Infections and Confocal Microscopy Intracellular Bacterial Community Enumeration

In preparation for bacterial inoculation into the bladder of mice, UPEC strains were grown in 20 ml of fresh LB broth at 37°C for about 24 h to induce the expression of type I fimbriae for invasion (Wright et al., 2005). Notably, 6-week-old female Balb/c mice were anesthetized and transurethrally inoculated with 10^7^ CFU of UPEC strains suspended in 50 μl PBS. The infection was performed 6 h before the mice were killed with anesthesia. The bladders were removed from the mice. For the assessment of bacterial titers, the bladders were homogenized in sterile PBS, and a suitable gradient dilution was plated for bacterial enumeration (Wright et al., 2005). Confocal microscopy IBC enumeration was performed following the reference from the laboratory of Scott J. Hultgren (Kostakioti et al., 2012). Mice were infected with fluorescent bacteria carrying the mCherry-plasmid, as described below for acute infections. Bladders were bisected, splayed, and fixed in 4% paraformaldehyde for 30 min. Fixed bladders were washed and counterstained for 20 min with 4′,6-diamidino-2-phenylindole (DAPI). For the Δ*yciR*pYciR strain, we used immunofluorescence to show the fluorescence and chose the anti-*E. coli* antibody (ab13627, 1:100) as the primary antibody and Alexa Fluor 594 conjugated secondary antibodies (ab150144, 1:200) following the methods from the Scott J. Hultgren’s lab (Mysorekar and Hultgren, 2006). We scanned the entire bladder tissues using a Zeiss LSM800 laser scanning confocal laser microscope (Carl Zeiss, United States) for total IBC enumeration manually and a single Z-section obtained by confocal laser microscopy demonstrated an IBC in superficial umbrella BEC of mice.

### Statistical Analysis

All the experiments were repeated in biological triplicates. GraphPad Prism version 6.0 was used for data analysis. The significance of mean values between two groups was evaluated by a two-tailed unpaired Student’s *t*-test or Mann–Whitney U test. The *p*-value < 0.05 was considered a significant difference for experiments. The graphical abstract was created using the BIORENDER tool^1^ with publication and licensing rights (Subscription: Student Plan; Agreement number: JR2407R2HN; Journal name: *Frontiers in Microbiology*).

## Data Availability Statement

The datasets presented in this study can be found in online repositories. The names of the repository/repositories and accession number(s) can be found below: https://www.ncbi.nlm.nih.gov/, GSE189294.

## Ethics Statement

The animal study was reviewed and approved by The Institutional Animal Care Committee at Nankai University and Tianjin Institute of Pharmaceutical Research New Drug Evaluation.

## Author Contributions

ZY, YD, and SZ: conceptualization. SZ, YF, JW, CQ, and WM: methodology. ZY and YD: validation and writing—review and editing. SZ, PL, and CY: formal analysis. SZ, YF, JW, and CQ: investigation. SZ, YF, PL, and YW: resources. WM, YD, YW, and CY: data curation. SZ, YF, and YD: writing—original draft preparation. ZY: funding acquisition. All authors contributed to the article and approved the submitted version.

## Conflict of Interest

The authors declare that the research was conducted in the absence of any commercial or financial relationships that could be construed as a potential conflict of interest.

## Publisher’s Note

All claims expressed in this article are solely those of the authors and do not necessarily represent those of their affiliated organizations, or those of the publisher, the editors and the reviewers. Any product that may be evaluated in this article, or claim that may be made by its manufacturer, is not guaranteed or endorsed by the publisher.

## Footnotes

1
https://biorender.com/
