# Supplementary material for: YciR, a Specific 3′-Phosphodiesterase, Plays a Role in the Pathogenesis of Uropathogenic Escherichia coli CFT073
Source: Front Microbiol. 2022 Jul 18;13:910906. doi: 10.3389/fmicb.2022.910906 (PMC9339999; doi:10.3389/fmicb.2022.910906)
Supplement: Supplementary file 1 [file Table_1.docx]

Supplementary Material


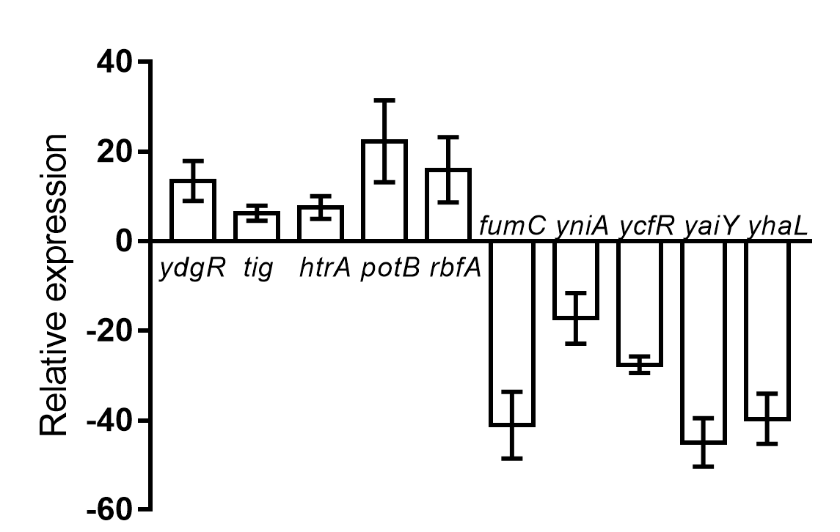


Figure S1. Confirmation of the RNA-seq results by qRT-PCR analysis, the expressions were normalized with the internal control gene gyrA. The tendencies of fold-change of qRT-PCR agreed with the results of RNA-seq. The error bars represent mean ± SD; n = 3.


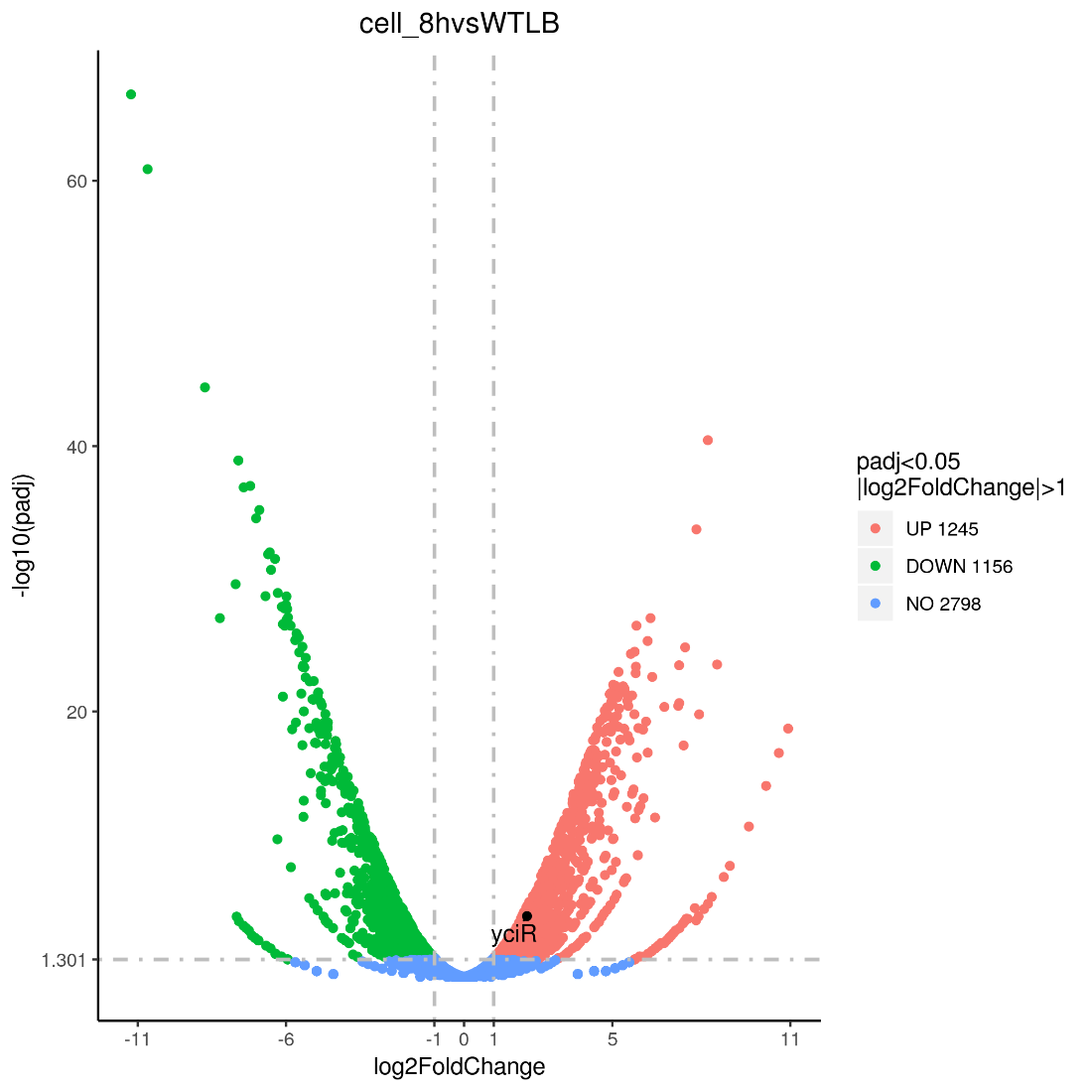


Figure S2. Volcano plots of different expression genes of up-regulated and down-regulated in 5637 cells compared to the CFT073 in LBbroth. The red dots represent up-regulated genes and green dots represent down-regulated genes.

Table S1. Strains and plasmids used in this study.

| Strain or plasmid | description |
| --- | --- |
| strains |  |
| WT | Wild-type stain uropathogenicescherichia coli CFT073 |
| Δ*yciR* | yciR coding sequence deletion mutant in Wild-type stain; Cm^R^ |
| Δ*yciR*/pYciR | The complement of YciR in yciR mutant strain |
| WT-mCherry | Wild-type stain containing a mCherryplasmid;Amp^R^ |
| Δ*yciR*-mCherry | ΔyciR strain containing a mCherryplasmid;Amp^R^, Cm^R^ |
| Δ*hupB* | hupB coding sequence deletion mutant in Wild-type stain; Cm^R^ |
| Δ*arcA* | arcA coding sequence deletion mutant in Wild-type stain; Cm^R^ |
| Δ*yebK* | yebK coding sequence deletion mutant in Wild-type stain; Cm^R^ |
| Δ*fucR* | fucR coding sequence deletion mutant in Wild-type stain; Cm^R^ |
| Δ*ygbI* | ygbI coding sequence deletion mutant in Wild-type stain; Cm^R^ |
| BL21 | for expressing protein |
| Plasmids |  |
| pKD3 | The template to amplify the cassette for l red recombination; Cm^R^ |
| pET28a | expression vector Km^R^ |
| pBluescript II-SK(+) | A high-copy T7 expression plasmid for gene complementary; Amp^R^ |
| pET28a-HupB | pET28a carrying hupB gene; Km^R^ |
| pETduet-mCherry | pETduet carrying mCherry; Amp^R^ |

Table S2. Primers used in this study

| Primer | Sequence (5’-3’) |
| --- | --- |
| *hupB* F *Eco*RI | CGGAATTCGTGAATAAATCTCAATTGATCGA |
| *hupB* R *Hin*dIII | CCAAGCTTTTATTAGTGGTGGTGGTGGTGGTGGTTTACCGCGTCTTTCAGT |
| pulldown-*yciR*-F | BIO-CGCGCCCAGTCGCGTAATCTCC |
| pulldown-*yciR*-R | BIO-GATGCATATCCTCCGGTTAACAG |
| RT *yciR* F | ATGAAAACCGTTAGGGAGTCCA |
| RT *yciR* R | GGTTCTGTGATTTCGGTGGT |
| RT *hupB* F | GTGAATAAATCTCAATTGATCG |
| RT *hupB* R | TACCAGTGCTACATCATCCC |
| RT *gyrA* F | ATGAGCGACCTTGCGAGAGAAA |
| RT *gyrA* R | GCTACCAAGAATACGCAACAG |
| FΔ*yciR* | CTGCGGACTCCGCTGTTAACCGGAGGATATGCATCGTGTAGGCTGGAGCTGCTTCG |
| RΔ*yciR* | GACGGAATATAGCGCTAAGTATATATATTCATCTACCATATGAATATCCTCCTTAG |
| JDFΔ*yciR* | CCATTCCTCATGGATGGGCCG |
| JDRΔ*yciR* | GTAATCGCCTGCCACAAGGATA |
| FΔ*hupB* | CAAGTGCGATATAAATTATAAAGAGGAAGAGAAGAGTGTAGGCTGGAGCTGCTTCG |
| RΔ*hupB* | CTTGAACTTCGTCACATCCCCACTGGGGACAACGCCATATGAATATCCTCCTTAG |
| JDFΔ*hupB* | GTGACTGCAAAATAGTGACCTC |
| JDRΔ*hupB* | GTTTGCAGCCGTGCGTAAGCT |
| FΔ*arcA* | CCTGTTTCGATTTAGTTGGCAATTTAGGTAGCAAACGTGTAGGCTGGAGCTGCTTCG |
| RΔ*arcA* | CTAAAAAGCGCCGTTTTTATTGACGGTGGTAAAGCCGACATATGAATATCCTCCTTAG |
| JDFΔ*arcA* | CTCTGCCGATAGCGCGCTGTTG |
| JDRΔ*arcA* | GTTAAAATGGTTAGGATGACAGCC |
| FΔ*yebK* | GCGGAAATCGTCATTACCCGTGAGTCTCTTTACATC |
| RΔ*yebK* | GATGTCGTATAAGATTAGGACAGTGACAGTCGTTTT |
| JDFΔ*yebK* | GCGATTACGGTAAAAGCGCGC |
| JDRΔ*yebK* | GTACAACGTTGGCACCCGCCGCG |
| FΔ*fucR* | CGCCCGCATGACGCGGGCGGTTATCGAATGATGGG |
| RΔ*fucR* | GGCCGGGGCGTCGTCTCCGGCCTGCTACCCTTTCCG |
| JDFΔ*fucR* | CCTGAAGTAGAACGACGTTATCG |
| JDRΔ*fucR* | CTTCAATCTCAAACTGCCGATG |
| FΔ*ygbI* | TTGCGCCGCGCACGTTTCGCAGGCAAATAGCGTAGA |
| RΔ*ygbI* | GTGAAGCATGAGTAACCCGGCGTTATTGCAGGTCA |
| JDFΔ*ygbI* | TTCCCATTGACCCTAAGCCAAC |
| JDRΔ*ygbI* | CTGGCCTGTTGATCGTGTGCAG |
| RT *ydgR* F | GTGTCCACTGCAAACCAAAAAC |
| RT *ydgR* R | AACCAATCGCCAGCACA |
| RT*tig* F | ATGCAAGTTTCAGTTGAAACCA |
| RT *tig* R | AGTTACGGCTCATCAGGTC |
| RT*htrA* F | ATGAAAAAAACCACATTAGCAC |
| RT *htrA* R | CTGCTGGGCTGTCGTTG |
| RT *potB* F | ATGAAGAACACAAGTAAGTTCC |
| RT *potB* R | TGGGCAGAAAGACAAACAACA |
| RT *rbfA* F | TTGTCAGGAGAATTTATTATGG |
| RT *rbfA* R | CGGAAACGGTGGTCATC |
| RT *fumC* F | ATGAATACAGTACGCAGCGAAA |
| RT *fumC* R | CGAGCGTTGAGTTTGTGC |
| RT *yniA* F | ATGTGGCAGGCAATCAGTCGTC |
| RT *yniA* R | ACTGATTGCCTGCCACAT |
| RT *ycfR* F | ATGAAAAACGTAAAAACCCTCA |
| RT *ycfR*R | GCAGCGATGAGGGTTTT |
| RT *yaiY* F | ATGGCTGATTTCACCCTGTCAA |
| RT*yaiY* R | GCCAGGCGTTGAAGATG |
| RT *yhaL* F | ATGATGATGAGTAAAAAATCGG |
| RT *yhaL*R  *yciR* F *Hin*dIII  *yciR* R *Bam*HI | CCACCACGGGCTTCACT  CCCAAGCTTGCAGCATTATTGCGCGCCCAGTCGCGTAATCTCC  CGCGGATCCTTATGCGCGCTTCAGATAGCGTTTATACCAGC |
